# Bis{μ-4,4′,6,6′-tetra-*tert*-butyl-2,2′-[*N*-(2-oxidoeth­yl)imino­dimethyl­ene]diphenolato}dialuminium(III)

**DOI:** 10.1107/S1600536810027212

**Published:** 2010-07-17

**Authors:** Stephanie L. Hemmingson, Alice J. Stevens, Joseph M. Tanski, Yutan D. Y. L. Getzler

**Affiliations:** a Departments of Chemistry & Biochemistry, Kenyon College, Gambier, OH 43214-9623, USA; b Department of Chemistry, Vassar College, 124 Raymond Ave., Box 406, Poughkeepsie, NY 12604-0744, USA

## Abstract

The title compound, [Al_2_(C_32_H_48_NO_3_)_2_], exists as a dimer with bridging ethoxide groups. It was isolated from a reaction mixture of the parent ligand and trimethyl­aluminium in tetra­hydro­furan. The geometry around the Al^III^ atom is a slightly distorted trigonal-bipyramid, typical of atrane derivatives.

## Related literature

For background to atranes, see: Voronkov & Baryshok (1982 ▶). For recent alumatrane work, see: Su *et al.* (2006 ▶) and references therein. For related structures and their activity in lactide polymerization, see: Johnson *et al.* (2009 ▶).
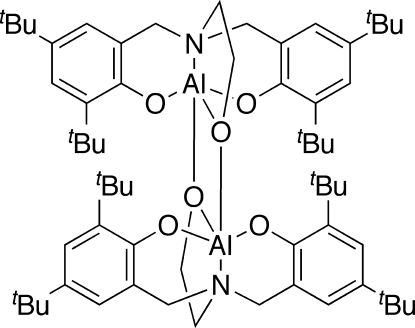


## Experimental

### 

#### Crystal data

[Al_2_(C_32_H_48_NO_3_)_2_]
*M* *_r_* = 1043.39
Monoclinic, 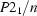

*a* = 13.385 (2) Å
*b* = 16.352 (3) Å
*c* = 14.141 (2) Å
β = 90.063 (2)°
*V* = 3095.1 (8) Å^3^
*Z* = 2
Mo *K*α radiation
μ = 0.10 mm^−1^
*T* = 125 K
0.26 × 0.16 × 0.09 mm

#### Data collection

Bruker APEXII CCD diffractometer
Absorption correction: multi-scan (*SADABS*; Bruker, 1999 ▶) *T*_min_ = 0.975, *T*_max_ = 0.991
42614 measured reflections
8485 independent reflections
5258 reflections with *I* > 2σ(*I*)
*R*_int_ = 0.076

#### Refinement

*R*[*F* ^2^ > 2σ(*F* ^2^)] = 0.048
*wR*(*F* ^2^) = 0.124
*S* = 1.02
8485 reflections
346 parameters
H-atom parameters constrained
Δρ_max_ = 0.28 e Å^−3^
Δρ_min_ = −0.30 e Å^−3^

### 

Data collection: *APEX2* (Bruker, 2007 ▶); cell refinement: *SAINT* (Bruker, 2007 ▶); data reduction: *SAINT*; program(s) used to solve structure: *SHELXS97* (Sheldrick, 2008 ▶); program(s) used to refine structure: *SHELXL97* (Sheldrick, 2008 ▶); molecular graphics: *Mercury* (Macrae *et al.*, 2008 ▶); software used to prepare material for publication: *enCIFer* (Allen *et al.*, 2004 ▶).

## Supplementary Material

Crystal structure: contains datablocks I, global. DOI: 10.1107/S1600536810027212/pk2252sup1.cif

Structure factors: contains datablocks I. DOI: 10.1107/S1600536810027212/pk2252Isup2.hkl

Additional supplementary materials:  crystallographic information; 3D view; checkCIF report

## Figures and Tables

**Fig. 1.**
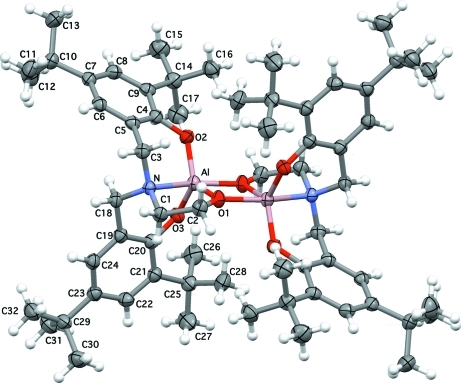
The molecular structure of the title compound with 50% probability displacement ellipsoids for non-H atoms. Unlabelled atoms are related to their labelled counterparts through an inversion operation (1-x, -y, 1-z).

**Fig. 2.**
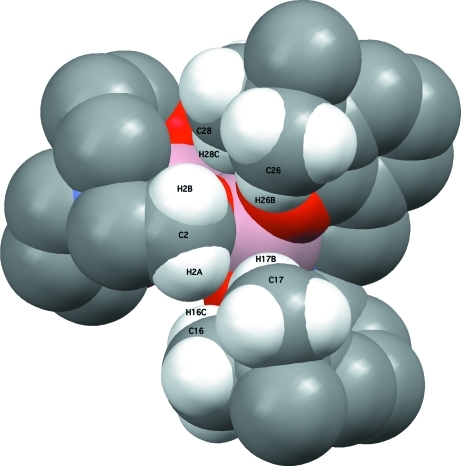
The molecular structure of the title compound, showing the crowded steric environment of the dimer. Atoms are drawn at their van der Waals radii with selected atoms removed to enhance clarity.

## supplementary crystallographic information

### Comment

Atranes are trigonal bipyramidal metal complexes featuring symmetric tripodal tetradentate ligands and a dative bond between the transannular chelated metal and the ligand nitrogen (Voronkov and Baryshok, 1982). They have been prepared with a range of metals and substantial recent work has been devoted to the potential catalytic activity of both atranes (Su *et al.*, 2006, and references therein) and their unsymmetric derivatives. In particular, previous work has shown that the monomeric alumatrane of tris(2-hydroxy-3,5-dimethylbenzyl)amine [N(CH_2_C_6_H_2_Me_2_OH)_3_] in the presence of 2-propanol shows activity for the melt polyermization of lactide (Johnson *et al.*, 2009). Replacing one hydroxybenzyl arm with a hydroxyethyl yields a dimeric complex which is inactive for polymerization. We surmised, erroneously, that perhaps increasing the bulk of the hydroxybenzyl substituent from methyl to *tert*-butyl would yield a compound capable of lactide polymerization. Reported here is the synthesis and characterization of the title compound.

The title compound (Fig. 1), exists as a dimer with bridging ethoxides. The molecule and its core {Al_2_O_2_} ring are centered on a point of crystallographic inversion. Aluminium has a slightly distorted trigonal-bipyramidal coordination geometry around the metal with equitorial O—Al—O angles ranging from 118.60 (6)° to 124.49 (5)° and an axial N—Al—O angle of 159.06 (5)°. Other bond lengths and angles are unremarkable. The Al—Al distance of 2.9022 (10) Å in the *tert*-butyl substituted title complex is barely distinguishable from the 2.8991 (7) Å A l—Al distance of the related methyl substituted compound (Johnson *et al.*, 2009). The dimer is crowded, as evidence by the proximity of the *tert*-butyl H atoms of one side of the dimer to the ethyloxy H atoms of the other side of the dimer (Fig. 2), which range from 2.523 (H2A—H17B) to 2.844 (H2B—H28C).

Attempts at melt polymerization of racemic lactide using the title compound proved fruitless, returning only starting materials. Indeed, variable temperature ^1^H NMR (-10 to 100 °C) of the title compound in toluene-d8 showed no evidence of the dimer breaking apart, a presumed necessity for polymerization activity. At high temperatures, however, the broad singlet at 2.441 resolves into a triplet (*J*=5.2 Hz) and the broad singlet at 3.302 resolves into two doublets (*J*=13 Hz). These dynamics are consistent with a sterically congested molecule in which rapid conformational equilibrium is achieved at elevated temperatures.

### Experimental

Ligand Synthesis: In a 250 ml round-bottom flask equipped with a magnetic stir-bar and a reflux condenser, 2,4-di-*tert*-butylphenol (Acros - 97%, 13.126 g, 63.62 mmol, 2.26 eq.) was dissolved in toluene. Formaldehyde (Fisher - 37% *w*/*w* in water, 5.2 ml, 64.1 mmol 2.27 eq) and ethanolamine (1.75 ml, 28.2 mmol, 1.00 eq.) were added and the reaction was refluxed overnight. The separated brown oil observed in the morning was isolated by rotary evaporator, further dried under high vacuum, and redissolved in 35 ml of 4:1 EtOH:H_2_O at 80 °C. Over several days, the temperature was gradually lowered to 51 °C at a rate no faster then 1 °C/min with the first crystals appearing after sitting at 63 °C overnight. The highly crystalline solid was isolated by vacuum filtration and washed with copious amounts of cold 4:1 EtOH:H_2_O. A residual yellow color in the product was removed by boiling the solid in 1:1 EtOH:MeOH yielding fine white crystals of the desired product (2.492 g, 5.01 mmol, 17.8% yield). ^1^H NMR (CDCl_3_, 300 MHz) δ: 1.297 (s, 18H, *t*-Bu), 1.423 (s, 18H, *t*-Bu), 2.754 (t, *J*=5.2 Hz, 2H, NCH_2_), 3.783 (s, 4H, ArCH_2_N), 3.891 (t, *J*=5.2 Hz, 2H, HOCH_2_), 6.926 (d, *J*=2.3 Hz, 2H, ArH), 7.240 (d, *J*=2.3 Hz, 2H, ArH); ^13^C NMR (CDCl_3_, 75 MHz) δ: 29.77, 31.80, 34.26, 35.03, 53.55, 57.83, 61.09, 121.75, 123.64, 125.14, 136.14, 141.22, 152.74.

Complex Synthesis: The complex may be synthesized by either the Johnson method (Johnson *et al.*, 2009) or as follows. Using standard Schlenk techniques, the free ligand was dissolved in THF and AlMe_3_ (Aldrich - 2.0 *M* in heptane, 1 equivalent) was added dropwise *via* syringe. Upon reaction completion, solvent was removed under high vacuum, quantitatively yielding a white powder which was pure by ^1^H NMR. Clear, colorless X-ray quality crystals may be obtained by layering hexanes on a concentrated THF solution of the complex or by cooling a concentrated solution of the complex in toluene.  ^1^H NMR (C_6_D_6_, 300 MHz) δ: 1.417 (s, 18H, *t*-Bu), 1.523 (s, 18H,*t*-Bu), 2.441 (br s, 2H, NCH_2_), 3.302 (br s, 4H, ArCH_2_N), 3.580 (t, *J*=5.8 Hz, 2H, OCH_2_), 6.782 (s, 2H, ArH), 7.509 (d, *J*=2.1 Hz, 2H, ArH); ^13^C NMR (C_6_D_6_, 75 MHz) δ: 29.83, 32.12, 34.36, 35.31, 53.08, 56.40, 57.63, 121.51, 124.33, 124.55, 138.09, 139.47, 155.95.

### Refinement

Hydrogen atoms on carbon were added geometrically and refined using a riding model. *U*_iso_ values for hydrogen atoms were assigned to be 1.20 times the *U*_eq_ value of the atom to which they are attached, except for hydrogen atoms on methyl carbon atoms, which were assigned a *U*_iso_ of 1.50 times the *U*_eq_ of the methyl carbon atom to which they are attached.

### Crystal data

**Table:**

[Al_2_(C_32_H_48_NO_3_)_2_]	*F*(000) = 1136
*M**_r_* = 1043.39	*D*_x_ = 1.120 Mg m^−^^3^
Monoclinic, *P*2_1_/*n*	Mo *K*α radiation, λ = 0.71073 Å
Hall symbol: -P 2yn	Cell parameters from 9619 reflections
*a* = 13.385 (2) Å	θ = 2.4–29.0°
*b* = 16.352 (3) Å	µ = 0.10 mm^−^^1^
*c* = 14.141 (2) Å	*T* = 125 K
β = 90.063 (2)°	Block, colorless
*V* = 3095.1 (8) Å^3^	0.26 × 0.16 × 0.09 mm
*Z* = 2	

### Data collection

**Table:**

Bruker APEXII CCD diffractometer	8485 independent reflections
Radiation source: fine-focus sealed tube	5258 reflections with *I* > 2σ(*I*)
graphite	*R*_int_ = 0.076
φ and ω scans	θ_max_ = 29.4°, θ_min_ = 1.9°
Absorption correction: multi-scan (*SADABS*; Bruker, 1999)	*h* = −18→18
*T*_min_ = 0.975, *T*_max_ = 0.991	*k* = −22→22
42614 measured reflections	*l* = −19→19

### Refinement

**Table:**

Refinement on *F*^2^	Primary atom site location: structure-invariant direct methods
Least-squares matrix: full	Secondary atom site location: difference Fourier map
*R*[*F*^2^ > 2σ(*F*^2^)] = 0.048	Hydrogen site location: inferred from neighbouring sites
*wR*(*F*^2^) = 0.124	H-atom parameters constrained
*S* = 1.02	*w* = 1/[σ^2^(*F*_o_^2^) + (0.054*P*)^2^ + 0.1736*P*] where *P* = (*F*_o_^2^ + 2*F*_c_^2^)/3
8485 reflections	(Δ/σ)_max_ = 0.001
346 parameters	Δρ_max_ = 0.28 e Å^−^^3^
0 restraints	Δρ_min_ = −0.30 e Å^−^^3^

### Special details

**Table:**

Geometry. All e.s.d.'s (except the e.s.d. in the dihedral angle between two l.s. planes) are estimated using the full covariance matrix. The cell e.s.d.'s are taken into account individually in the estimation of e.s.d.'s in distances, angles and torsion angles; correlations between e.s.d.'s in cell parameters are only used when they are defined by crystal symmetry. An approximate (isotropic) treatment of cell e.s.d.'s is used for estimating e.s.d.'s involving l.s. planes.
Refinement. Refinement of *F*^2^ against ALL reflections. The weighted *R*-factor *wR* and goodness of fit *S* are based on *F*^2^, conventional *R*-factors *R* are based on *F*, with *F* set to zero for negative *F*^2^. The threshold expression of *F*^2^ > σ(*F*^2^) is used only for calculating *R*-factors(gt) *etc*. and is not relevant to the choice of reflections for refinement. *R*-factors based on *F*^2^ are statistically about twice as large as those based on *F*, and *R*- factors based on ALL data will be even larger.An extinction parameter (EXTI in SHELXL-97) refined to zero and was removed from the refinement.

### Fractional atomic coordinates and isotropic or equivalent isotropic displacement parameters (Å^2^)

**Table:**

	*x*	*y*	*z*	*U*_iso_*/*U*_eq_	
Al	0.48567 (3)	0.00620 (3)	0.60145 (3)	0.01834 (12)	
N	0.45295 (9)	0.11682 (8)	0.66499 (9)	0.0186 (3)	
O1	0.48322 (8)	0.06840 (6)	0.49393 (7)	0.0207 (2)	
O2	0.59510 (8)	−0.01009 (6)	0.66678 (7)	0.0210 (2)	
O3	0.37507 (8)	−0.03952 (6)	0.63921 (7)	0.0211 (2)	
C1	0.40568 (12)	0.16764 (9)	0.59021 (11)	0.0216 (3)	
H1A	0.4095	0.2262	0.6078	0.026*	
H1B	0.3344	0.1526	0.5834	0.026*	
C2	0.46004 (12)	0.15345 (9)	0.49708 (11)	0.0217 (3)	
H2A	0.4169	0.1688	0.4430	0.026*	
H2B	0.5219	0.1865	0.4944	0.026*	
C3	0.54955 (11)	0.15462 (9)	0.69665 (11)	0.0201 (3)	
H3A	0.5361	0.2098	0.7225	0.024*	
H3B	0.5943	0.1609	0.6414	0.024*	
C4	0.62348 (11)	0.02194 (10)	0.75104 (11)	0.0198 (3)	
C5	0.60123 (11)	0.10398 (9)	0.77066 (11)	0.0194 (3)	
C6	0.62328 (11)	0.13734 (10)	0.85915 (11)	0.0210 (3)	
H6A	0.6072	0.1929	0.8714	0.025*	
C7	0.66820 (11)	0.09106 (10)	0.92947 (11)	0.0212 (3)	
C8	0.69347 (11)	0.01025 (10)	0.90613 (11)	0.0223 (3)	
H8A	0.7260	−0.0218	0.9530	0.027*	
C9	0.67405 (11)	−0.02613 (9)	0.81904 (11)	0.0202 (3)	
C10	0.68331 (12)	0.12239 (10)	1.03076 (11)	0.0253 (4)	
C11	0.65734 (15)	0.21287 (11)	1.04082 (13)	0.0375 (5)	
H11A	0.5869	0.2213	1.0244	0.056*	
H11B	0.6996	0.2451	0.9983	0.056*	
H11C	0.6688	0.2302	1.1063	0.056*	
C12	0.61407 (15)	0.07270 (13)	1.09584 (13)	0.0392 (5)	
H12A	0.5446	0.0797	1.0753	0.059*	
H12B	0.6213	0.0921	1.1611	0.059*	
H12C	0.6322	0.0147	1.0925	0.059*	
C13	0.79151 (13)	0.10983 (13)	1.06342 (13)	0.0380 (5)	
H13A	0.7988	0.1292	1.1286	0.057*	
H13B	0.8366	0.1407	1.0221	0.057*	
H13C	0.8083	0.0516	1.0604	0.057*	
C14	0.70394 (12)	−0.11522 (10)	0.79799 (12)	0.0247 (4)	
C15	0.76285 (14)	−0.15419 (11)	0.87969 (13)	0.0345 (4)	
H15A	0.7803	−0.2106	0.8632	0.052*	
H15B	0.7218	−0.1540	0.9370	0.052*	
H15C	0.8241	−0.1228	0.8910	0.052*	
C16	0.77086 (13)	−0.11864 (11)	0.70923 (12)	0.0302 (4)	
H16A	0.7892	−0.1755	0.6961	0.045*	
H16B	0.8315	−0.0864	0.7202	0.045*	
H16C	0.7344	−0.0961	0.6550	0.045*	
C17	0.61021 (13)	−0.16820 (10)	0.78223 (13)	0.0316 (4)	
H17A	0.6304	−0.2234	0.7631	0.047*	
H17B	0.5689	−0.1437	0.7325	0.047*	
H17C	0.5718	−0.1712	0.8411	0.047*	
C18	0.38553 (11)	0.10632 (9)	0.74813 (11)	0.0205 (3)	
H18A	0.3706	0.1608	0.7751	0.025*	
H18B	0.4210	0.0743	0.7972	0.025*	
C19	0.28857 (11)	0.06402 (9)	0.72497 (11)	0.0202 (3)	
C20	0.28792 (11)	−0.00799 (9)	0.67177 (11)	0.0197 (3)	
C21	0.19640 (12)	−0.04808 (9)	0.65258 (11)	0.0206 (3)	
C22	0.11042 (12)	−0.01534 (10)	0.69270 (11)	0.0227 (3)	
H22A	0.0488	−0.0424	0.6810	0.027*	
C23	0.10895 (12)	0.05482 (10)	0.74907 (11)	0.0218 (3)	
C24	0.19948 (12)	0.09479 (10)	0.76260 (11)	0.0223 (3)	
H24A	0.2009	0.1441	0.7982	0.027*	
C25	0.19244 (12)	−0.12450 (10)	0.58915 (12)	0.0248 (4)	
C26	0.25483 (14)	−0.19357 (10)	0.63343 (13)	0.0317 (4)	
H26A	0.2279	−0.2072	0.6959	0.047*	
H26B	0.3244	−0.1756	0.6399	0.047*	
H26C	0.2520	−0.2420	0.5927	0.047*	
C27	0.08587 (13)	−0.15707 (11)	0.57662 (15)	0.0380 (5)	
H27A	0.0440	−0.1146	0.5480	0.057*	
H27B	0.0585	−0.1722	0.6384	0.057*	
H27C	0.0870	−0.2053	0.5354	0.057*	
C28	0.23320 (14)	−0.10343 (11)	0.49074 (12)	0.0317 (4)	
H28A	0.1948	−0.0579	0.4640	0.048*	
H28B	0.2272	−0.1513	0.4494	0.048*	
H28C	0.3036	−0.0876	0.4960	0.048*	
C29	0.01071 (12)	0.08307 (10)	0.79368 (12)	0.0259 (4)	
C30	−0.06841 (13)	0.09706 (11)	0.71690 (13)	0.0346 (4)	
H30A	−0.0467	0.1413	0.6749	0.052*	
H30B	−0.1320	0.1120	0.7466	0.052*	
H30C	−0.0772	0.0468	0.6801	0.052*	
C31	−0.02621 (13)	0.01629 (11)	0.86174 (13)	0.0336 (4)	
H31A	0.0219	0.0097	0.9135	0.050*	
H31B	−0.0328	−0.0355	0.8275	0.050*	
H31C	−0.0913	0.0321	0.8877	0.050*	
C32	0.02250 (13)	0.16235 (11)	0.85008 (14)	0.0354 (4)	
H32A	0.0470	0.2057	0.8083	0.053*	
H32B	0.0703	0.1538	0.9017	0.053*	
H32C	−0.0423	0.1783	0.8764	0.053*	

### Atomic displacement parameters (Å^2^)

**Table:**

	*U*^11^	*U*^22^	*U*^33^	*U*^12^	*U*^13^	*U*^23^
Al	0.0206 (2)	0.0179 (2)	0.0165 (2)	0.00029 (19)	0.00106 (18)	−0.00016 (19)
N	0.0186 (7)	0.0212 (7)	0.0160 (6)	−0.0004 (5)	0.0006 (5)	0.0005 (5)
O1	0.0273 (6)	0.0164 (5)	0.0183 (5)	0.0026 (4)	0.0024 (5)	0.0002 (4)
O2	0.0211 (6)	0.0213 (6)	0.0205 (6)	0.0009 (4)	−0.0022 (4)	−0.0023 (4)
O3	0.0205 (6)	0.0198 (6)	0.0229 (6)	0.0009 (4)	0.0035 (5)	−0.0022 (5)
C1	0.0251 (8)	0.0189 (8)	0.0208 (8)	0.0044 (6)	−0.0022 (7)	0.0012 (6)
C2	0.0288 (9)	0.0154 (8)	0.0207 (8)	0.0025 (6)	−0.0012 (7)	0.0018 (6)
C3	0.0213 (8)	0.0179 (8)	0.0211 (8)	−0.0025 (6)	0.0009 (6)	0.0000 (6)
C4	0.0177 (8)	0.0235 (8)	0.0183 (8)	−0.0030 (6)	0.0022 (6)	−0.0002 (6)
C5	0.0178 (8)	0.0203 (8)	0.0202 (8)	−0.0017 (6)	0.0016 (6)	0.0018 (6)
C6	0.0190 (8)	0.0210 (8)	0.0229 (8)	−0.0023 (6)	0.0025 (6)	−0.0012 (7)
C7	0.0183 (8)	0.0254 (8)	0.0201 (8)	−0.0022 (6)	0.0013 (6)	−0.0005 (6)
C8	0.0184 (8)	0.0257 (9)	0.0227 (8)	−0.0005 (6)	−0.0009 (6)	0.0041 (7)
C9	0.0161 (8)	0.0209 (8)	0.0235 (8)	−0.0006 (6)	0.0013 (6)	0.0031 (6)
C10	0.0254 (9)	0.0310 (9)	0.0195 (8)	0.0011 (7)	−0.0007 (7)	−0.0023 (7)
C11	0.0502 (12)	0.0365 (11)	0.0258 (10)	0.0045 (9)	−0.0069 (9)	−0.0102 (8)
C12	0.0445 (12)	0.0489 (12)	0.0241 (10)	−0.0020 (9)	0.0067 (8)	0.0020 (9)
C13	0.0316 (10)	0.0525 (12)	0.0300 (10)	0.0046 (9)	−0.0082 (8)	−0.0122 (9)
C14	0.0265 (9)	0.0211 (8)	0.0265 (9)	0.0017 (7)	−0.0007 (7)	0.0013 (7)
C15	0.0380 (11)	0.0272 (10)	0.0383 (11)	0.0064 (8)	−0.0041 (8)	0.0023 (8)
C16	0.0274 (9)	0.0282 (9)	0.0351 (10)	0.0058 (7)	0.0016 (8)	−0.0031 (8)
C17	0.0342 (10)	0.0220 (9)	0.0385 (11)	−0.0040 (7)	0.0006 (8)	0.0008 (8)
C18	0.0232 (8)	0.0207 (8)	0.0176 (8)	−0.0006 (6)	0.0021 (6)	−0.0034 (6)
C19	0.0212 (8)	0.0206 (8)	0.0188 (8)	−0.0014 (6)	0.0007 (6)	0.0004 (6)
C20	0.0205 (8)	0.0208 (8)	0.0178 (8)	0.0006 (6)	0.0012 (6)	0.0025 (6)
C21	0.0226 (8)	0.0203 (8)	0.0188 (8)	−0.0011 (6)	−0.0001 (6)	0.0003 (6)
C22	0.0196 (8)	0.0236 (8)	0.0250 (8)	−0.0019 (6)	−0.0012 (6)	−0.0004 (7)
C23	0.0207 (8)	0.0242 (8)	0.0206 (8)	0.0018 (6)	0.0000 (6)	0.0012 (6)
C24	0.0256 (9)	0.0212 (8)	0.0200 (8)	0.0019 (7)	0.0001 (7)	−0.0028 (6)
C25	0.0241 (9)	0.0222 (8)	0.0280 (9)	−0.0028 (7)	0.0004 (7)	−0.0054 (7)
C26	0.0380 (10)	0.0193 (9)	0.0377 (10)	−0.0025 (7)	0.0008 (8)	−0.0019 (7)
C27	0.0314 (10)	0.0326 (10)	0.0500 (12)	−0.0071 (8)	0.0028 (9)	−0.0169 (9)
C28	0.0367 (10)	0.0339 (10)	0.0245 (9)	−0.0014 (8)	−0.0023 (8)	−0.0077 (8)
C29	0.0195 (8)	0.0284 (9)	0.0299 (9)	0.0021 (7)	0.0009 (7)	−0.0049 (7)
C30	0.0273 (10)	0.0335 (10)	0.0430 (11)	0.0067 (8)	−0.0050 (8)	−0.0038 (8)
C31	0.0231 (9)	0.0435 (11)	0.0340 (10)	0.0028 (8)	0.0071 (8)	0.0005 (8)
C32	0.0260 (9)	0.0383 (11)	0.0418 (11)	0.0033 (8)	0.0035 (8)	−0.0141 (9)

### Geometric parameters (Å, °)

**Table:**

Al—O3	1.7428 (11)	C15—H15A	0.9800
Al—O2	1.7513 (11)	C15—H15B	0.9800
Al—O1	1.8295 (11)	C15—H15C	0.9800
Al—O1^i^	1.8661 (11)	C16—H16A	0.9800
Al—N	2.0668 (14)	C16—H16B	0.9800
Al—Al^i^	2.9022 (10)	C16—H16C	0.9800
N—C1	1.4858 (19)	C17—H17A	0.9800
N—C18	1.4928 (19)	C17—H17B	0.9800
N—C3	1.5011 (19)	C17—H17C	0.9800
O1—C2	1.4255 (18)	C18—C19	1.506 (2)
O1—Al^i^	1.8661 (11)	C18—H18A	0.9900
O2—C4	1.3554 (18)	C18—H18B	0.9900
O3—C20	1.3564 (18)	C19—C20	1.397 (2)
C1—C2	1.523 (2)	C19—C24	1.400 (2)
C1—H1A	0.9900	C20—C21	1.415 (2)
C1—H1B	0.9900	C21—C22	1.391 (2)
C2—H2A	0.9900	C21—C25	1.539 (2)
C2—H2B	0.9900	C22—C23	1.397 (2)
C3—C5	1.503 (2)	C22—H22A	0.9500
C3—H3A	0.9900	C23—C24	1.390 (2)
C3—H3B	0.9900	C23—C29	1.530 (2)
C4—C5	1.402 (2)	C24—H24A	0.9500
C4—C9	1.414 (2)	C25—C27	1.533 (2)
C5—C6	1.396 (2)	C25—C28	1.534 (2)
C6—C7	1.386 (2)	C25—C26	1.538 (2)
C6—H6A	0.9500	C26—H26A	0.9800
C7—C8	1.404 (2)	C26—H26B	0.9800
C7—C10	1.534 (2)	C26—H26C	0.9800
C8—C9	1.392 (2)	C27—H27A	0.9800
C8—H8A	0.9500	C27—H27B	0.9800
C9—C14	1.540 (2)	C27—H27C	0.9800
C10—C11	1.527 (2)	C28—H28A	0.9800
C10—C13	1.533 (2)	C28—H28B	0.9800
C10—C12	1.539 (2)	C28—H28C	0.9800
C11—H11A	0.9800	C29—C32	1.530 (2)
C11—H11B	0.9800	C29—C30	1.533 (2)
C11—H11C	0.9800	C29—C31	1.538 (2)
C12—H12A	0.9800	C30—H30A	0.9800
C12—H12B	0.9800	C30—H30B	0.9800
C12—H12C	0.9800	C30—H30C	0.9800
C13—H13A	0.9800	C31—H31A	0.9800
C13—H13B	0.9800	C31—H31B	0.9800
C13—H13C	0.9800	C31—H31C	0.9800
C14—C15	1.536 (2)	C32—H32A	0.9800
C14—C17	1.541 (2)	C32—H32B	0.9800
C14—C16	1.544 (2)	C32—H32C	0.9800
			
O3—Al—O2	118.90 (6)	C17—C14—C16	109.57 (14)
O3—Al—O1	118.60 (6)	C14—C15—H15A	109.5
O2—Al—O1	122.49 (5)	C14—C15—H15B	109.5
O3—Al—O1^i^	97.54 (5)	H15A—C15—H15B	109.5
O2—Al—O1^i^	95.43 (5)	C14—C15—H15C	109.5
O1—Al—O1^i^	76.51 (5)	H15A—C15—H15C	109.5
O3—Al—N	93.55 (5)	H15B—C15—H15C	109.5
O2—Al—N	94.67 (5)	C14—C16—H16A	109.5
O1—Al—N	82.59 (5)	C14—C16—H16B	109.5
O1^i^—Al—N	159.06 (5)	H16A—C16—H16B	109.5
O3—Al—Al^i^	112.72 (4)	C14—C16—H16C	109.5
O2—Al—Al^i^	113.54 (4)	H16A—C16—H16C	109.5
O1—Al—Al^i^	38.70 (3)	H16B—C16—H16C	109.5
O1^i^—Al—Al^i^	37.81 (3)	C14—C17—H17A	109.5
N—Al—Al^i^	121.28 (4)	C14—C17—H17B	109.5
C1—N—C18	111.57 (12)	H17A—C17—H17B	109.5
C1—N—C3	110.36 (12)	C14—C17—H17C	109.5
C18—N—C3	109.49 (11)	H17A—C17—H17C	109.5
C1—N—Al	105.68 (9)	H17B—C17—H17C	109.5
C18—N—Al	111.73 (9)	N—C18—C19	113.77 (12)
C3—N—Al	107.89 (9)	N—C18—H18A	108.8
C2—O1—Al	121.34 (9)	C19—C18—H18A	108.8
C2—O1—Al^i^	135.16 (9)	N—C18—H18B	108.8
Al—O1—Al^i^	103.50 (5)	C19—C18—H18B	108.8
C4—O2—Al	129.66 (10)	H18A—C18—H18B	107.7
C20—O3—Al	132.22 (10)	C20—C19—C24	120.18 (14)
N—C1—C2	109.07 (12)	C20—C19—C18	120.58 (13)
N—C1—H1A	109.9	C24—C19—C18	119.11 (14)
C2—C1—H1A	109.9	O3—C20—C19	119.88 (13)
N—C1—H1B	109.9	O3—C20—C21	120.22 (14)
C2—C1—H1B	109.9	C19—C20—C21	119.89 (14)
H1A—C1—H1B	108.3	C22—C21—C20	117.38 (14)
O1—C2—C1	106.25 (12)	C22—C21—C25	121.49 (14)
O1—C2—H2A	110.5	C20—C21—C25	121.13 (14)
C1—C2—H2A	110.5	C21—C22—C23	124.13 (15)
O1—C2—H2B	110.5	C21—C22—H22A	117.9
C1—C2—H2B	110.5	C23—C22—H22A	117.9
H2A—C2—H2B	108.7	C24—C23—C22	116.86 (14)
N—C3—C5	112.12 (12)	C24—C23—C29	123.41 (14)
N—C3—H3A	109.2	C22—C23—C29	119.72 (14)
C5—C3—H3A	109.2	C23—C24—C19	121.44 (15)
N—C3—H3B	109.2	C23—C24—H24A	119.3
C5—C3—H3B	109.2	C19—C24—H24A	119.3
H3A—C3—H3B	107.9	C27—C25—C28	107.76 (15)
O2—C4—C5	118.98 (14)	C27—C25—C26	107.27 (14)
O2—C4—C9	121.10 (14)	C28—C25—C26	109.92 (14)
C5—C4—C9	119.93 (14)	C27—C25—C21	112.39 (13)
C6—C5—C4	120.43 (14)	C28—C25—C21	109.52 (13)
C6—C5—C3	120.36 (14)	C26—C25—C21	109.93 (14)
C4—C5—C3	119.16 (14)	C25—C26—H26A	109.5
C7—C6—C5	121.37 (15)	C25—C26—H26B	109.5
C7—C6—H6A	119.3	H26A—C26—H26B	109.5
C5—C6—H6A	119.3	C25—C26—H26C	109.5
C6—C7—C8	116.73 (14)	H26A—C26—H26C	109.5
C6—C7—C10	122.96 (14)	H26B—C26—H26C	109.5
C8—C7—C10	120.15 (14)	C25—C27—H27A	109.5
C9—C8—C7	124.44 (15)	C25—C27—H27B	109.5
C9—C8—H8A	117.8	H27A—C27—H27B	109.5
C7—C8—H8A	117.8	C25—C27—H27C	109.5
C8—C9—C4	116.94 (14)	H27A—C27—H27C	109.5
C8—C9—C14	121.81 (14)	H27B—C27—H27C	109.5
C4—C9—C14	121.25 (14)	C25—C28—H28A	109.5
C11—C10—C13	108.47 (15)	C25—C28—H28B	109.5
C11—C10—C7	112.38 (14)	H28A—C28—H28B	109.5
C13—C10—C7	111.10 (13)	C25—C28—H28C	109.5
C11—C10—C12	108.58 (15)	H28A—C28—H28C	109.5
C13—C10—C12	108.57 (15)	H28B—C28—H28C	109.5
C7—C10—C12	107.64 (14)	C32—C29—C23	112.48 (14)
C10—C11—H11A	109.5	C32—C29—C30	108.27 (14)
C10—C11—H11B	109.5	C23—C29—C30	110.26 (14)
H11A—C11—H11B	109.5	C32—C29—C31	107.96 (14)
C10—C11—H11C	109.5	C23—C29—C31	108.69 (13)
H11A—C11—H11C	109.5	C30—C29—C31	109.09 (14)
H11B—C11—H11C	109.5	C29—C30—H30A	109.5
C10—C12—H12A	109.5	C29—C30—H30B	109.5
C10—C12—H12B	109.5	H30A—C30—H30B	109.5
H12A—C12—H12B	109.5	C29—C30—H30C	109.5
C10—C12—H12C	109.5	H30A—C30—H30C	109.5
H12A—C12—H12C	109.5	H30B—C30—H30C	109.5
H12B—C12—H12C	109.5	C29—C31—H31A	109.5
C10—C13—H13A	109.5	C29—C31—H31B	109.5
C10—C13—H13B	109.5	H31A—C31—H31B	109.5
H13A—C13—H13B	109.5	C29—C31—H31C	109.5
C10—C13—H13C	109.5	H31A—C31—H31C	109.5
H13A—C13—H13C	109.5	H31B—C31—H31C	109.5
H13B—C13—H13C	109.5	C29—C32—H32A	109.5
C15—C14—C9	112.35 (14)	C29—C32—H32B	109.5
C15—C14—C17	107.02 (14)	H32A—C32—H32B	109.5
C9—C14—C17	110.38 (13)	C29—C32—H32C	109.5
C15—C14—C16	107.38 (14)	H32A—C32—H32C	109.5
C9—C14—C16	110.03 (13)	H32B—C32—H32C	109.5
			
O3—Al—N—C1	−94.79 (10)	C6—C7—C8—C9	−1.8 (2)
O2—Al—N—C1	145.82 (9)	C10—C7—C8—C9	173.74 (15)
O1—Al—N—C1	23.61 (9)	C7—C8—C9—C4	−1.4 (2)
O1^i^—Al—N—C1	27.2 (2)	C7—C8—C9—C14	179.92 (14)
Al^i^—Al—N—C1	24.58 (11)	O2—C4—C9—C8	−175.28 (13)
O3—Al—N—C18	26.74 (10)	C5—C4—C9—C8	4.2 (2)
O2—Al—N—C18	−92.66 (10)	O2—C4—C9—C14	3.4 (2)
O1—Al—N—C18	145.14 (10)	C5—C4—C9—C14	−177.13 (14)
O1^i^—Al—N—C18	148.75 (14)	C6—C7—C10—C11	−8.1 (2)
Al^i^—Al—N—C18	146.11 (8)	C8—C7—C10—C11	176.65 (15)
O3—Al—N—C3	147.15 (9)	C6—C7—C10—C13	−129.89 (17)
O2—Al—N—C3	27.76 (10)	C8—C7—C10—C13	54.9 (2)
O1—Al—N—C3	−94.45 (9)	C6—C7—C10—C12	111.36 (17)
O1^i^—Al—N—C3	−90.84 (17)	C8—C7—C10—C12	−63.84 (19)
Al^i^—Al—N—C3	−93.48 (9)	C8—C9—C14—C15	−4.4 (2)
O3—Al—O1—C2	88.09 (12)	C4—C9—C14—C15	176.98 (15)
O2—Al—O1—C2	−92.74 (12)	C8—C9—C14—C17	114.95 (16)
O1^i^—Al—O1—C2	179.41 (14)	C4—C9—C14—C17	−63.65 (19)
N—Al—O1—C2	−1.91 (11)	C8—C9—C14—C16	−124.00 (16)
Al^i^—Al—O1—C2	179.41 (14)	C4—C9—C14—C16	57.40 (19)
O3—Al—O1—Al^i^	−91.31 (6)	C1—N—C18—C19	61.03 (17)
O2—Al—O1—Al^i^	87.85 (7)	C3—N—C18—C19	−176.50 (12)
O1^i^—Al—O1—Al^i^	0.0	Al—N—C18—C19	−57.03 (15)
N—Al—O1—Al^i^	178.68 (6)	N—C18—C19—C20	47.9 (2)
O3—Al—O2—C4	−74.98 (13)	N—C18—C19—C24	−136.28 (14)
O1—Al—O2—C4	105.86 (13)	Al—O3—C20—C19	−33.4 (2)
O1^i^—Al—O2—C4	−176.69 (12)	Al—O3—C20—C21	147.05 (12)
N—Al—O2—C4	21.68 (13)	C24—C19—C20—O3	−176.94 (14)
Al^i^—Al—O2—C4	148.83 (11)	C18—C19—C20—O3	−1.1 (2)
O2—Al—O3—C20	114.92 (13)	C24—C19—C20—C21	2.6 (2)
O1—Al—O3—C20	−65.88 (14)	C18—C19—C20—C21	178.45 (14)
O1^i^—Al—O3—C20	−144.59 (13)	O3—C20—C21—C22	176.23 (14)
N—Al—O3—C20	17.61 (14)	C19—C20—C21—C22	−3.3 (2)
Al^i^—Al—O3—C20	−108.54 (13)	O3—C20—C21—C25	−4.2 (2)
C18—N—C1—C2	−161.42 (12)	C19—C20—C21—C25	176.21 (14)
C3—N—C1—C2	76.61 (15)	C20—C21—C22—C23	1.0 (2)
Al—N—C1—C2	−39.79 (14)	C25—C21—C22—C23	−178.53 (15)
Al—O1—C2—C1	−19.85 (16)	C21—C22—C23—C24	2.0 (2)
Al^i^—O1—C2—C1	159.33 (11)	C21—C22—C23—C29	−176.98 (15)
N—C1—C2—O1	38.79 (17)	C22—C23—C24—C19	−2.8 (2)
C1—N—C3—C5	−178.55 (12)	C29—C23—C24—C19	176.16 (15)
C18—N—C3—C5	58.27 (16)	C20—C19—C24—C23	0.5 (2)
Al—N—C3—C5	−63.54 (13)	C18—C19—C24—C23	−175.34 (14)
Al—O2—C4—C5	−38.36 (19)	C22—C21—C25—C27	0.5 (2)
Al—O2—C4—C9	141.13 (12)	C20—C21—C25—C27	−179.07 (15)
O2—C4—C5—C6	175.61 (13)	C22—C21—C25—C28	120.19 (16)
C9—C4—C5—C6	−3.9 (2)	C20—C21—C25—C28	−59.34 (19)
O2—C4—C5—C3	−1.9 (2)	C22—C21—C25—C26	−118.94 (17)
C9—C4—C5—C3	178.61 (14)	C20—C21—C25—C26	61.53 (19)
N—C3—C5—C6	−121.61 (15)	C24—C23—C29—C32	3.1 (2)
N—C3—C5—C4	55.91 (18)	C22—C23—C29—C32	−178.02 (15)
C4—C5—C6—C7	0.6 (2)	C24—C23—C29—C30	124.02 (17)
C3—C5—C6—C7	178.04 (14)	C22—C23—C29—C30	−57.1 (2)
C5—C6—C7—C8	2.2 (2)	C24—C23—C29—C31	−116.44 (17)
C5—C6—C7—C10	−173.17 (14)	C22—C23—C29—C31	62.5 (2)

Symmetry codes: (i) −*x*+1, −*y*, −*z*+1.
